# Gender differences in the effects of emotion induction on intertemporal decision-making

**DOI:** 10.1371/journal.pone.0299591

**Published:** 2024-03-20

**Authors:** Eleonora Fiorenzato, Patrizia Bisiacchi, Giorgia Cona

**Affiliations:** 1 Department of Neuroscience, University of Padua, Padua, Italy; 2 Department of General Psychology, University of Padua, Padua, Italy; 3 Padova Neuroscience Center (PNC), University of Padua, Padua, Italy; Julius-Maximilians-Universität Würzburg, GERMANY

## Abstract

‘Good things come to those who wait’ is a popular saying, which goes along with numerous daily life decisions requiring trade-offs between immediate-small and later-larger rewards; however, some individuals have a tendency to prefer sooner rewards while discounting the value of delayed rewards, known as delay discounting. The extant literature indicates that emotions and gender can modulate intertemporal choices, but their interplay remains hitherto poorly investigated. Here, 308 participants were randomized to different conditions, inducing distinct emotions–fear, joy, a neutral state–through standardized movie clips, and then completed a computerized delay discounting task for hypothetical money rewards. Following the induction of fear, women discount the future steeper than men, thus preferring immediate-smaller rewards rather than larger-delayed ones. Also, women were more prone to choose immediate rewards when in a fearful condition than when in a positive state of joy/happiness. By contrast, men were unaffected by their emotional state when deciding on monetary rewards. Our findings provide evidence that fear can trigger different intertemporal choices according to gender, possibly reflecting the adoption of different evolutionary strategies.

## Introduction

Decision-making is more than a purely deliberative cognitive process. Emotions–affective states that activate motivational and cognitive predispositions–can indeed modulate decisions [1, 2], as also corroborated by neuroimaging evidence, given their underlying and overlapping brain networks [3]. Research confirms that a reciprocal relationship between decision-making and emotions exists [1]: namely, emotional states have an impact on judgment and decision-making, and similarly decisions can modulate emotions by increasing or reducing positive/negative states.

In everyday decision-making, individuals face trade-offs between short versus long-term benefits or costs; and depending on many factors, they can choose to wait for the larger delayed reward, but in other situations they prefer the smaller immediate reward. The phenomenon, known as delay discounting (DD), refers to the typical tendency to prefer a sooner reward than a later one, even if the delayed reward magnitude is larger–resulting in high discount rates in economic terms [4, 5]. How much the value of delayed rewards is discounted is modulated by several factors and, among these, emotions represent a pervasive determinant [6, 7]. However, to date, findings on the impact of discrete emotions on DD remain controversial [8]. This possibly derives from the fact that most studies adopt an emotional valence-based approach, focusing on positive versus negative valence, rather than exploring the distinct influences of specific emotions, such as anger, fear, or joy, on intertemporal choices [1, 9]. In addition, the variety of adopted methods to induce emotions–such as static pictures (e.g., emotional facial expressions), movie clips, autobiographical recall, imagination techniques–have further contributed to increase the heterogeneity in the results of the literature, although movie clips have been indicated as the most effective method among the standardized procedures [10, 11]. A further reason is the relative absence of investigations exploring inter-individual differences in the relationship between DD and emotions. In particular, gender’s role within the interplay of temporal discounting and emotions is overlooked. This is striking, considering that gender differences in DD choices [5, 12], as well as in emotional processing have been largely reported [13], with the latter being supported also by numerous psychophysiological and neuroimaging evidence, pointing to grounded biological and evolutionary differences [13, 14].

Understanding inter-individual differences in DD as well as the factors influencing DD and its underlying mechanisms is of particular relevance, as this phenomenon has been consistently linked to various unhealthy behaviors [15, 16]. Namely, the extent to which individuals discount the value of delayed rewards turns out to be associated with alcohol and drug dependence [17, 18], gambling and eating disorders [19, 20], tobacco use [21], risky sexual behaviors [22], financial hardships [23], and reduced subjective wellbeing [24]. Although it has been argued that discounting rates seem to reflect a quite stable personality trait [16], there is also growing evidence that DD can be modulated by contextual/situational factors [25]. Steeper discounting rates were found linked to higher perception of stress, lower life satisfaction, anxiety and depressive moods [15].

Considering that everyday decisions are emotion-imbued choices [1] and that DD represents an effective measure of decision-making tendencies, in the present investigation, we examine how distinct emotions (fear vs. joy) and gender interact to influence intertemporal choices. These choices involve decisions between sooner (usually smaller) rewards and later (usually larger) ones, which are common and have significant consequences in daily life.

### The influence of emotions on delay discounting

Numerous studies have explored the role of emotions in DD. However, inconsistent findings have left the exact interaction between emotions and DD yet to be fully understood [8]. Overall, both positive and negative emotional valences can decrease/increase DD rates, and this heterogeneity among investigations is possibly due to research paradigm and method differences in inducing affective states. In particular, some studies–using short movie clips or words to induce a positive/neutral affect–demonstrated that positive emotions reduce the preference for present over future outcomes, suggesting that positive emotional states promote cognitive flexibility, higher level of thinking and a more future-oriented decision-making [26, 27]. While others, using similar emotion induction methods, did not confirm a reduction of DD rates in the context of positive emotions [28, 29]. Indeed, they found that extroverted individuals, when in a positive mood, exhibited an increased preference for immediate rewards [29]. Additional evidence from studies that induced positive emotional states found a link between positive emotions and reduced future discounting. For instance, this reduction was observed when participants experienced gratitude through autobiographical recall [30, 31], inducing positive nostalgia (i.e., reminiscing past positive events) [32] or manipulating the emotional valence of episodic prospection [33].

In line with these observations, Guan and colleagues (2015) examined the continuum of positive, neutral, and negative emotions induced by affective pictures. They found that in the positive condition, delay discounting was attenuated, whereas in the negative condition, it was characterized by a more myopic decision-making behavior [34]. Furthermore, it should be noted that evidence on the impact of negative emotions on DD has also been mixed, with some studies reporting a general null effect [29], others showing an increased preference for delayed rewards in the context of fearful conditions [35], and others reporting the opposite scenario, with negative emotional state being linked to steeper DD [28, 33, 34, 36–38].

A further reason of such variability across studies might stem from the different underlying theoretical background to define the emotional phenomena. Prior research described and measured emotions categorically with specific and discrete emotional states (i.e., sadness, anger, happiness and fear), or dimensionally, usually across two continuums: valence (negative-positive) and arousal [39]. More recently, empirical evidence has extended dimensional models of emotion, recommending that four major dimensions are required to support adequate discrimination of distinct emotions: valence, arousal, power/control, and unpredictability [9]. Likewise, another well-grounded multidimensional theoretical framework is the appraisal-tendency framework (ATF), which assumes that specific emotions give rise to specific cognitive and motivational processes, leading to different effects on decision-making behaviors [40]. Namely, each emotion is related to specific appraisals and these will differently affect judgment and decision-making [1, 9, 40–42]. Of note, to define the patterns of appraisal tendencies underlying different emotions, the ATF mainly builds on the theory of Smith & Ellsworth (1985), which distinguished six cognitive dimensions of emotion: pleasantness, certainty, attentional activity, control, anticipated effort and responsibility [41].

Given that progress has been made to define and understand the dimensions that support emotional phenomena, investigating the relationship between emotions and decision-making, building on multidimensional framework (e.g., the ATF), can improve the comprehension of the different intertemporal choices.

### Gender and decision-making

Among the inter-individual differences that can potentially influence decision-making, gender differences in discounting might be probably one of the most relevant factors [43, 44]. However, gender’s role remains yet underexplored in the majority of the published studies, wherein these differences are frequently not discussed, despite the inclusion of mixed samples [45].

Much of the research on gender differences in decision-making has focused on impulsive behaviors and the responsiveness to gains and losses, which are critical aspects of the decision-making process. One established finding is that females are more risk-avoidant (i.e., less impulsive) than males (e.g., for a review see [46]), possibly due to females’ higher sensitivity to losses [47]. These observations challenge the simplistic view that explains gender differences solely in terms of impulsivity, and they encourage further exploration of other underlying mechanisms in decision-making [48]. In support of this concept, more recently, Byrne & Worthy (2015) highlighted that reward-sensitivity and information processing better describes the complex interplay between gender and decision-making [49]. Notably, the authors found distinctive gender decision-making styles. While males showed a cognitive bias towards maximizing long-term benefits by using a more global-selective information processing style, females had a tendency to maximize either immediate or long-term rewards in different situations by using more detailed-comprehensive processors of information [49].

Focusing on DD choices, gender’s role is poorly defined. Using hypothetical DD measures to examine how gender affects attention to immediate versus long-term rewards, there is evidence for females showing steeper discount rates–that is, a preference for immediate smaller rewards [12, 50–52]. By contrast, other studies revealed males were higher discounters [5, 37, 44], and others reported no gender effects [53–56].

These contradictory findings can be possibly explained by various methodological differences between studies to assess DD. Such as, differences in the magnitude of the reward (smaller vs. larger ones: $10 vs. $1000) and its nature (hypothetical vs. real reward), the different delays adopted, the use of questionnaires rather than DDT-computerized versions, the applied statistical analysis method to estimate the discounting index (either *k* or area under the curve). Other possible differences are represented by the sample size and its demographical features (e.g., age, education, socio-economic status, race), and situational factors induced during the experimental design (e.g., showing appealing/not-appealing pictures or inducing a failure/success state before the DDT execution) [44, 54].

Although results from the literature are mixed, in the review of Weafer and de Wit (2014), the authors conclude that the majority of human studies seem to converge on greater impulsive choice for hypothetical rewards in women, whereas men may show greater impulsive choice for actual rewards [43]. Specifically, these gender differences can be explained by a greater sensitivity for punishment/losses and effortful control in women, as opposed to a greater sensitivity to reward in men [43]. These different attitudes toward risks were further confirmed by other evidence [57], that identified men optimism as a prominent modulating factor of this difference. Indeed, a general positive disposition of men results in different approaches to achieve rewards and information processing styles during decision-making, which can also be explained with an evolutionary framework [5, 37, 57].

### Current study

Based on the lack of studies specifically designed to examine the influence of gender in the interplay between emotions and decision-making, in the current study, we directly compared male-versus-female DD rates, following the induction of specific emotions. We employed a validated set of emotional movie clips to elicit discrete emotions with a more ecological and clear-cut method [58]. A major limitation of past research has been the use of static picture to elicit emotions (e.g., emotional facial expressions) [59], although videoclips have been repeatedly confirmed as more effective than static pictures to experimentally induce emotions [60, 61]. Movies are more dynamic and engage viewers through multiple senses, including both auditory and visual experiences. Movies were, therefore, more effective in eliciting greater emotional involvement among participants. Furthermore, using this set of standardized movies allowed us to induce three distinct emotions–fear, joy, a neutral state–each with a different emotional valence (negative, positive, neutral).

Based on the multidimensional models of emotion, fear (being negative in valence) can be described by the appraisals of low certainty-control, whereas happiness/joy (positive valence) is characterized by the appraisals of high certainty-control [1]. Therefore, we would anticipate observing opposite effects on decision-making, as fear and joy vary along multiple dimensions, beyond their valence, which can influence cognitive appraisals and decision-making.

In addition, it is worth considering that DD is a multifaceted phenomenon, which can be modulated by different factors, such as time-preference (e.g., choosing between current consumption and restraint/future consumption), risk-preference (e.g., choosing between a less certain, riskier option and a more certain option with less risk), loss/punishment sensitivity (e.g., the degree to which an individual can tolerate loss/punishment) or reward sensitivity (e.g., the degree to which an individual’s behavior is motivated by reward-relevant stimuli). Hence, interpreting different intertemporal choices can be analyzed from different perspective.

From a risk-preference perspective, a delayed but larger reward can be perceived as riskier compared to an immediate but smaller reward because the future is always uncertain, in contrast to the known present [62, 63]. Additionally, individual differences in reward sensitivity or time preference can also account for this behavior [64].

Overall, given these premises, we can hypothesize that fear–with low certainty-control–will produce a tendency to perceive negative events as unpredictable, leading to a pessimistic risk assessment and consequently to perceive an overall higher risk [41, 42]. Thus, a negative/fear condition is more likely to induce individuals to choose an immediate reward, as this is considered a less risky option. By contrast, in its positive valence, joy/happiness produces a tendency to perceive events as predictable, leading to relatively optimistic risk assessments and choices [41, 42], and so to wait for the delayed larger rewards, despite being considered riskier and more uncertain.

In addition, we investigated potential gender differences in intertemporal choices as a function of distinct induced emotions. Considering that previous studies have reported gender differences in reward/loss sensitivity [43], with women having a tendency to prefer immediate-reward while men maximizing future rewards [49, 65]. On the other side, gender differences in optimism (usually higher in men) has been described to affect the willingness to take risks. Hence, we can expect that different approaches to achieve the rewards–as an expression of gender differences–may interact with distinct induced emotions, leading to different intertemporal choices.

Here, to achieve this aim, we designed a web-based study that was spread nationwide to reach a large cohort and ensure an adequate representation across Italy. Our experiment included a standardized emotion induction using validated movie clips [58], followed by a computerized behavioral task of hypothetical monetary rewards, the delay discounting task (DDT), to objectively assess differences in decision-making tendencies. Of note, considering that the data collection occurred during COVID-19 pandemic, wherein higher level of depression, anxiety and stress has been reported worldwide [66] as well as in the Italian populace [67]; we took into consideration also the psychological distress of the study’s participants at that time, given that anxious-depressive symptoms have been associated with differences in intertemporal decision-making [68, 69].

## Material and methods

### Participants and procedures

**Fig 1:**
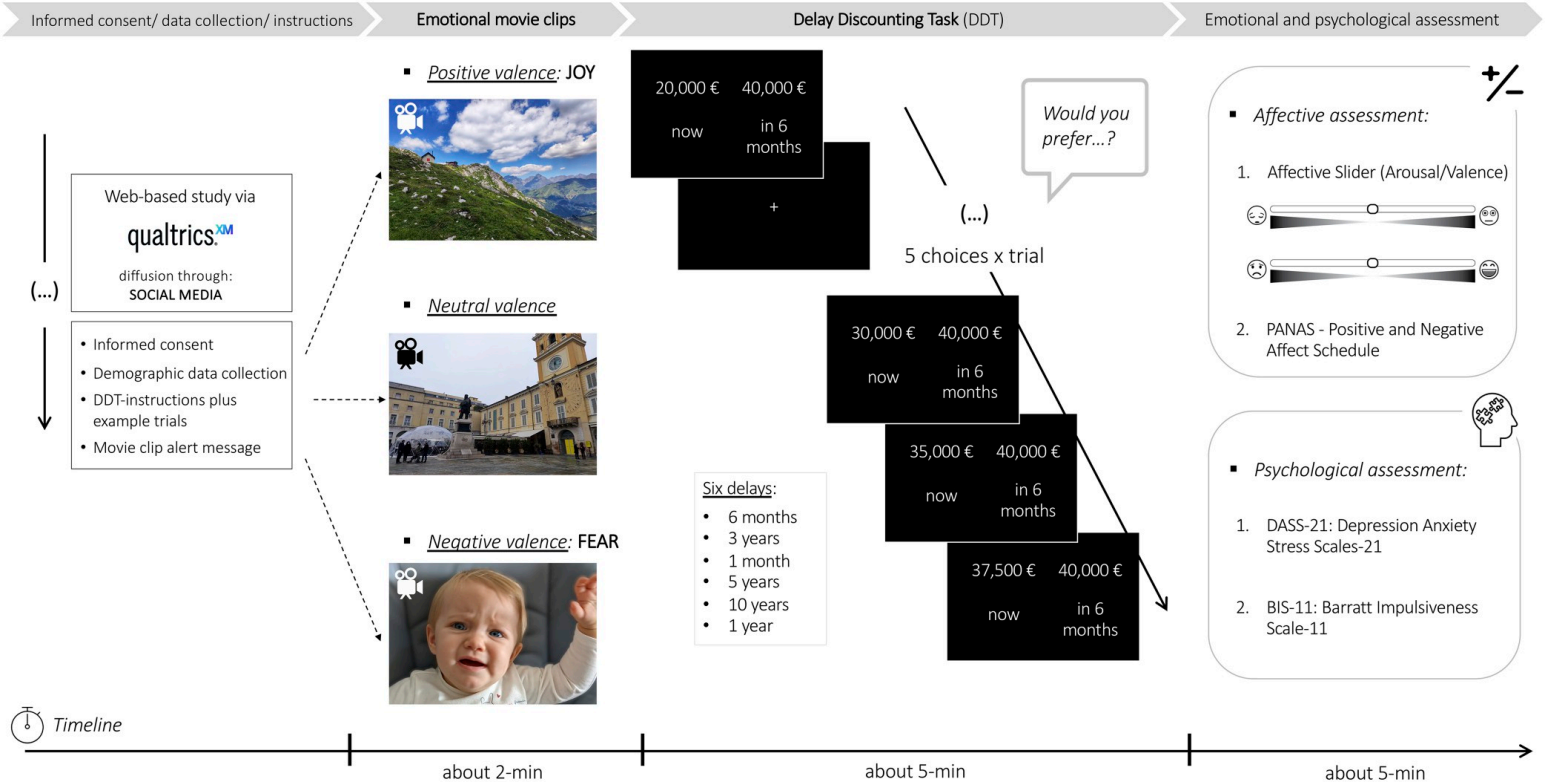
Summary of the study design including the emotional induction phase, the experimental task and the affective/psychological assessment. The guardian of the individual pictured in this figure has provided written informed consent (as outlined in PLOS consent form) to publish their image alongside the manuscript.

An anonymous online survey was shared through various platforms and mainstream social media from May 7 to July 15, 2021 (70 days). To reach a large cohort, a snowball sampling method was used. Furthermore, participants were encouraged to share and invite new respondents among their social contacts. Participation was voluntary and without compensation. The study was structured in five main parts described in detail hereafter: informed consent, sociodemographic data collection, emotional state induction through a videoclip, experimental task, and self-reported questionnaires completion. The research workflow is shown in Fig 1.

Namely, a brief introduction described the research aims, followed by an informed consent request ensuring data confidentiality. The study took about 15 minutes. Sociodemographic, health status and lifestyle features were collected. Among the sociodemographic variables we collected the age, the biological sex, educational level and occupation of the participants. Concerning the sex/gender terminology in the current manuscript, for simplicity the term ‘gender’ was adopted instead of using ‘sex’–following the American Psychological Association (APA) guidelines [70]–since the term ‘gender’ is able to capture the sociocultural dimension of the sex/gender concept. In fact, ‘gender’ refers to a series of norms and expectations, for both females and males, which are modulated by several factors as well as psychological processes [71]. Noteworthy, the entire research community is nowadays moving toward a more holistic perspective that considers sex (i.e., biology) and gender (i.e., culture) as inseparable and intertwined [70, 71]. Then, the instructions and example trials of the experimental task–a hypothetical money reward DDT–were presented, informing the participants that they were going to perform the DDT immediately after viewing a movie clip. At the end of the task, we assessed the affective experience (i.e., arousal/valence levels and positive/negative affection) elicited by the movie clip, together with other psychological dimensions (i.e., depression, anxiety, stress, and impulsivity). We considered the responses as eligible if participants: completed the entire study, were over 18 years-old, and had no history of neurologic disorders. Among a total of 328 responses via Qualtrics’ platform, 308 were eligible based on our inclusion criteria (n = 11 did not complete the survey; n = 9 reported a neurological disorder). In this regard, our final sample was in accordance with the required sample size that was *apriori* determined through G*power (version 3.1) [72], wherein we ran ANCOVA models (main effects and interactions). Given that no previous published works investigated exactly our research question (for a review see [8]), the expected effect size was determined by selecting the smallest reported effect size from comparable studies: small-to-intermediate effect (i.e., Cohen’s *f* = 0.18 [37]). Thus, with a desired power of 0.80, alpha probability of .05 and a small-to-intermediate effect (*f* = 0.18), the required total sample was equal to 301 participants. The current study was conducted in accordance with the Helsinki Declaration and approved by the ethical committee of the School of Psychology University of Padua, Padua, Italy.

### Emotional stimuli

**Fig 2:**
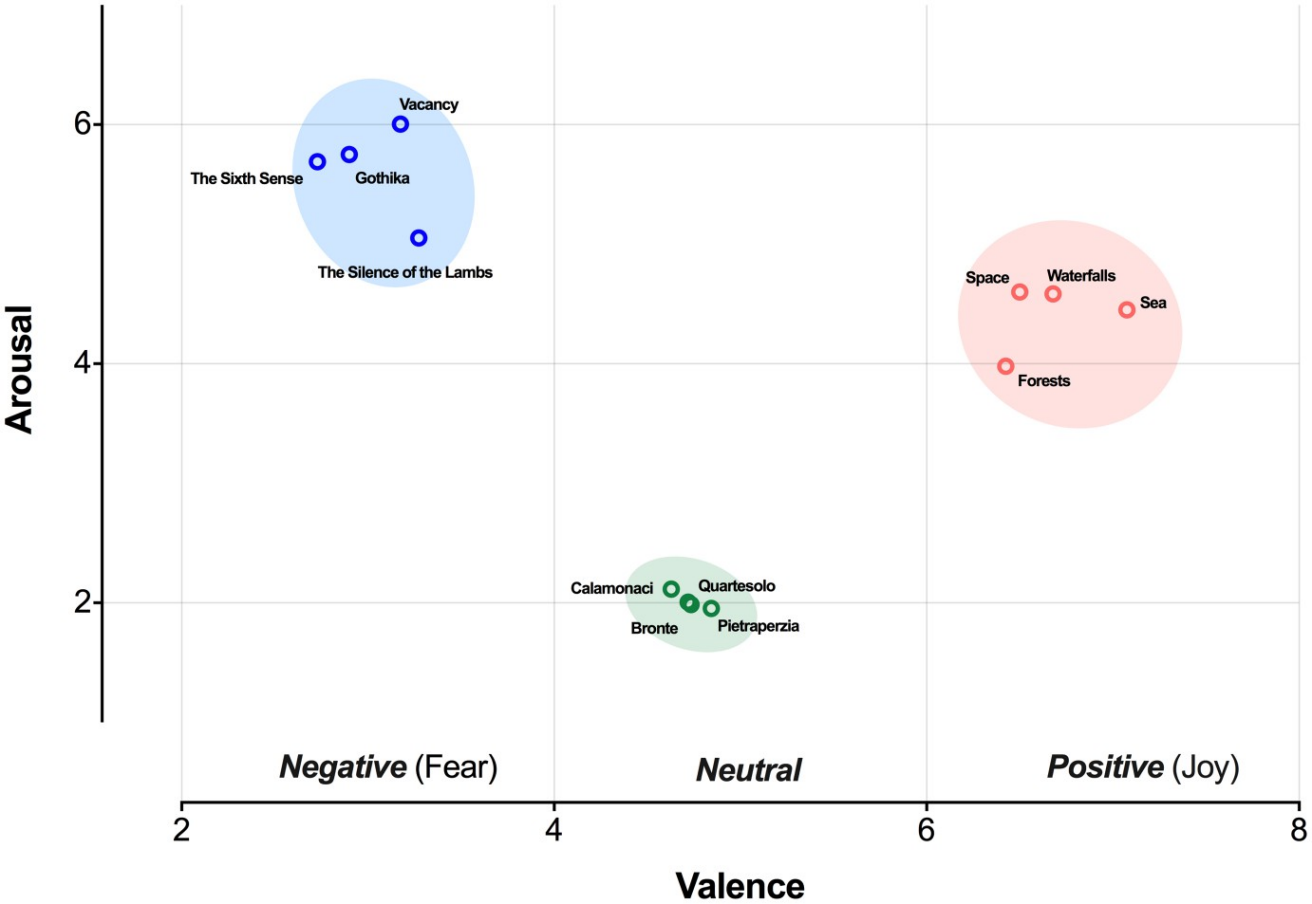
Distribution of the standardized movie clips according to the arousal scores and emotional valence categorization (based on the validation study of Maffei and Angrilli, 2019).

The stimuli were 12 standardized movie clips, selected from the ‘E-MOVIE’ dataset [58]. In the validation study [58], the clips were categorized and validated according to their arousal and valence. In Fig 2, the distribution of the movies considering their arousal and emotional valence categorization are displayed (for further details about ‘EMOVIE’, refer to the original work of Maffei and Angrilli, 2019) [58].

About the emotional valence, four clips were selected for each of the three conditions: Negative (thrilling scenes of anticipated threat), Positive (scenery eliciting joy/happiness–with stunning views of various natural landscapes), and Neutral (scenes drawn from urban documentaries). The movie clips were selected to elicit distinct emotions within the negative-positive valence spectrum: Fear (within the Negative category) to induce unpleasant and aversive responses; Joy (within the Positive category) to elicit pleasant and appetitive responses; and the Neutral condition was placed in the middle of this continuum.

The emotional condition labels–Fear, Neutral, Joy/Happiness–will be used throughout the manuscript.

As established in the published literature [58, 73], these movies differ both in terms of valence and arousal levels, with ‘Fear movies’ having the highest arousal levels, followed by ‘Joy movies’ and Neutral category (see Fig 2). Notably, to avoid the ‘movie-effect’ rather than the emotion effect, we selected four standardized clips for each category. These 2-minute clips were designed [58] to be homogeneous in terms of emotion induction within each category, and to be more arousing in their second part. Using Qualtrics software, participants were randomized into one of three emotional conditions–Fear, Neutral, Joy–and randomly assigned to watch a 2-minute movie. Subsequently participants were redirected to the experimental task.

### Behavioral measures

#### Decision-making: Delay discounting task

In the DDT, participants were required to choose between two virtual money amounts in each trial: a hypothetical smaller money amount to be given immediately (e.g., €20,000 today) versus a larger amount at a later timepoint (e.g., €40,000 after 3 years). For each of the six delays (1 month, 6 months, 1 year, 3 years, 5 years, 10 years), participants had to perform five choices and this process allowed us to obtain the indifference point for each delay, namely the money amount at which an individual was equally likely to choose a smaller reward sooner rather than a delayed larger reward. In sum, the indifference point corresponds to the unshown sixth choice of immediate amount. The order of the presented six delays was kept constant, as previously described [74]. The delayed amount was fixed at €40,000, whereas the immediate amount started at €20,000 but then it was modulated by the previous response (increasing vs. decreasing). Previous studies reported a ‘reward magnitude effect’–that is, steeper discounting for smaller rewards (e.g., $1,000) rather than larger ones (e.g., $10,000) [52, 75]. Here, given that the delays were mainly in a range of years (until 10 years), we adopted the larger amounts (€40,000) [15] to ask the participants for more plausible intertemporal decisions as well as to avoid the steepest discounting effect observed when using smaller rewards [52]. Finally, to quantify the subjective degree of DD, the area under the curve (AUC) was calculated according to the indifference points at each delay. The DDT AUC ranges from 0 to 1 (high vs. no discount rate, respectively). This is considered as a reliable measure of future-oriented behaviors requiring self-control in cases of lower discount (i.e., preference for larger delayed rewards) versus immediate-oriented behaviors, that are more impulsive, in cases of higher discount rate (i.e., preference for smaller earlier rewards) [76, 77]. Although this task was based on hypothetical rewards, a good correspondence with real ones has been demonstrated [78] as well as with non-monetary outcomes [75]. The DDT was designed using OpenSesame v.3.3.6 with the OSWeb v.1.3.11 extension [79] to run the task online.

As in previous studies, the emotion induction effect was evaluated after the DD task by using validated scales to assess emotional states and impulsivity. Valence and arousal were also recorded to obtain a self-reported measure of the emotional induction, although the clips were already validated for these dimensions [58].

#### Affective experience

The affective experience was evaluated by means of the i) Affective Slider [80] as well as by the ii) Positive and Negative Affect Schedule (PANAS) [81]. The Affective Slider (see Fig 1) allows to digitally assess the perceived level of arousal (low to high) and valence (negative to positive–measured as displeasure to pleasure). The 2-sliders position on the screen (top vs. bottom) randomly varied across participants to prevent order-effects.

Participants then completed the 10-item PANAS scales, measuring their affective experience valence: positive (PANAS-P) versus negative (PANAS-N), with higher scores indicating higher levels of affect. Cronbach’s alphas were consistent with the scale validated in Italian [81]: for PANAS-P the score was equal to .82 and .77 for PANAS-N. Further, to enhance the precision of PANAS in the context of parametric statistics, we applied the published ordinal-to-interval conversions scores [82].

#### Depression, anxiety, stress and impulsivity assessment

The Depression Anxiety Stress Scales-21 (DASS-21) was used to assess presence of depression, anxiety and stress by means of the validated Italian version, showing excellent psychometric properties. DASS-21 is widely used in clinical and research to assess psychopathological symptoms, given its ability with three 7-item subscales: depression (DASS-D), anxiety (DASS-A) and stress (DASS-S). Higher total scores indicate higher severity in terms of symptoms, and the published cut-off scores for each subscale were adopted to identify presence of clinically significant disturbances [83]. DASS-21 internal consistency in the current samples was excellent for the total score (α = .93) and high for the subscales (DASS-D α = .90; DASS-A α = .79; DASS-S α = .87).

Self-reported impulsivity was assessed using the Barratt Impulsiveness Scale-11 (BIS-11), translated into Italian [84]. BIS-11 consists of 30 items, rated on a self-report 5-point Likert scale, wherein higher total scores suggest more elevated impulsivity levels. The internal consistency of the BIS-11 in the current study was high with Cronbach’s α = .81.

### Statistical analysis

Descriptive analyses were performed for all behavioral measures. To investigate the independent and interactive effects of Gender and Emotional Condition (Fear, Neutral, Joy) on DD, a two-way ANCOVA was run. This model included the DDT AUC as the dependent variable, while Emotional Condition and Gender as between-subject factors. The potential confounding effect of age and education [51], of arousal (due to its difference between emotional stimuli and genders) [58, 85], and of psychological disturbances (DASS-21) was controlled by including those variables as covariates. We conducted further ANOVA models to assess potential differences between groups in the affective experience (arousal, valence, PANAS-N and PANAS-P), and psychological dimensions (DASS-21 and its subscales, BIS-11). Based on the movie clip validation study [58], we expected to find significant differences in arousal level between the three emotional conditions. Thus, when analyzing the affective experience outcomes, we further consider entering arousal level as covariate into ANOVAs to exclude possible confounding effect, if this difference was confirmed. Effect sizes were estimated using partial eta squared (ηp^2^) and 95 percent confidence interval (CI) reported when appropriate. Statistical significance threshold was set at p < .05 and False Discovery Rate (FDR) was used to correct for multiple testing. Statistical analyses were performed using SPSS Statistic, release version 24.0 (Chicago, IL, USA).

## Results

### Participant sociodemographic and lifestyle related features

Total sample (N = 308) sociodemographic and lifestyle characteristics are shown in Table 1. Notably, the sample was balanced across experimental conditions, with about 33 percent of the sample in the Fear, Neutral or Joy movie clip condition. However, the sample was unbalanced for gender (f/m) with a majority of female participants in each condition: in the Fear (63/41), Neutral (67/36) or Joy (65/36). To balance the dataset according to gender, we applied a random undersampling technique, where the largest classes (i.e., the female ones) were randomly trimmed until their size was equal to the corresponding smallest class (i.e., male one). We obtained a balanced dataset for gender [female/male: Fear (41/41), Neutral (36/36) and Joy (36/36) condition] and given that the results were comparable to the imbalanced one, here we reported the results of the total sample, whereas the results run in the reduced sample (n = 226) are reported in the S1 File

**Table 1:** Total sample (N = 308) sociodemographic and lifestyle features.

		Group	n	%
**Age**	**Mean (SD):** 33.73(14.39)	18–24	124	40.26
		25–40	100	32.47
	**Min–max:** 18–76	>40	84	27.27
**Gender**		Female	195	63.31
		Male	113	36.69
**Education**		Middle school	18	5.84
		High school	136	44.16
		Bachelor’s degree	92	29.87
		Master’s degree	44	14.29
		PhD/ postgraduate	18	5.84
**Occupation**		Teacher/Researcher	16	5.20
		Medical staff	12	3.90
		Clerk	47	15.26
		Freelancer	24	7.79
		Unemployed	15	4.87
		Student	115	37.34
		Retired	15	4.87
		Manager	14	4.55
		Workman/Artisan	18	5.84
		Householder	3	0.97
		Other	29	9.41
**Smoke**		No	203	65.91
		Yes	105	34.09
**Psychotropic drugs**		No	293	95.13
		Yes	15	4.87
**Alcohol consumption**		Never	170	55.19
		Occasionally	112	36.36
		Usually	26	8.44
**Cannabis consumption**		Never	163	52.92
		Occasionally	124	40.26
		Usually	21	6.82
**Other substances**		Never	286	92.86
		Occasionally	20	6.49
		Usually	2	0.65
**Emotional movie clip**		Negative	104	33.77
		Neutral	103	33.44
		Positive	101	32.79

Of note, the six experimental subgroups (classified for Gender × Emotional Movie clip), did not differ in terms of age, education as well as on the other sociodemographic variables (data not shown).

### Gender and induced emotion effects on delay discounting

**Fig 3:**
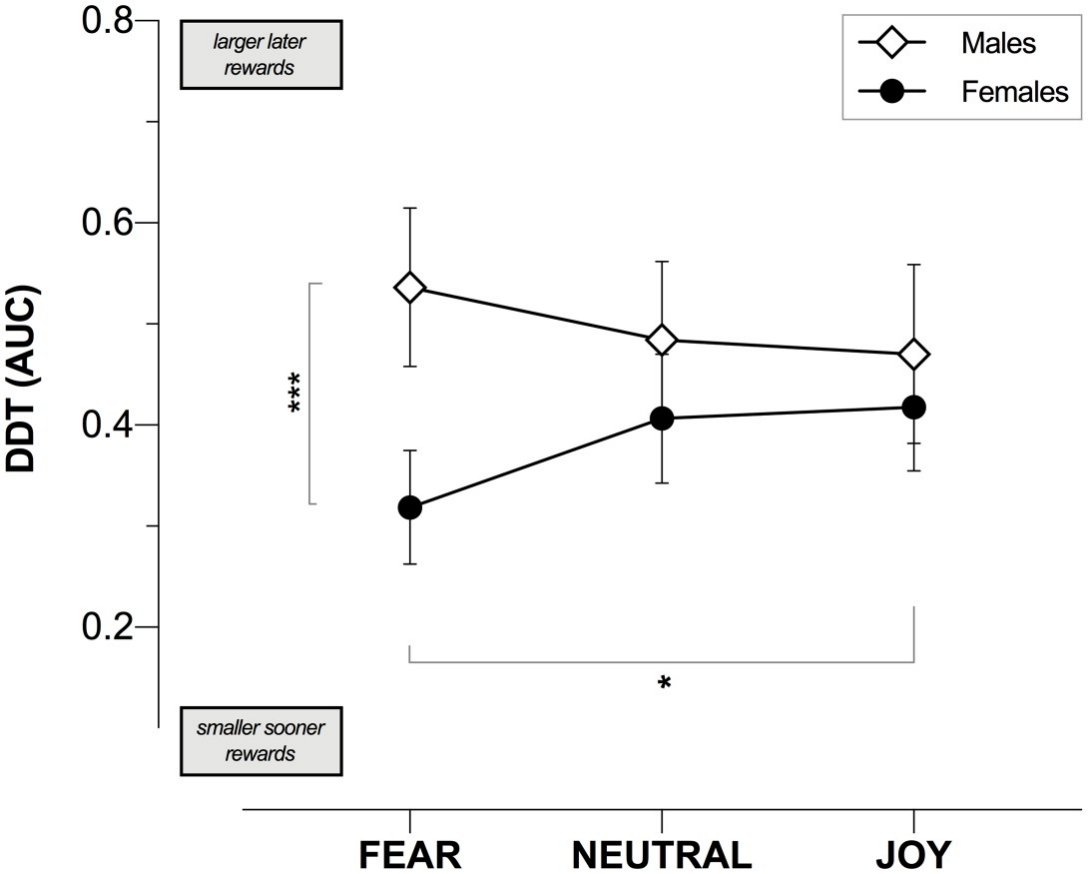
Gender and induced emotion effect on delay discounting, as measured by the area under the curve (AUC) of the delay discounting task (DDT). Error bars represent the 95% confidence interval of the mean. *** p < .001; * p < .05.

ANCOVA results showed a significant Gender × Emotional Condition interaction on DD (*F*_2,297_ = 3.96, *p* = .020, η_p_^2^ = .03; Fig 3). Post-hoc analysis revealed that in the Fear condition, women (0.32 ± 0.22) showed a statistically significant higher DD than men (0.54 ± 0.25) (*t*_297_ = -4.50, *p*_FDR_ < .001, *d* = -0.91, CI [-1.32, -0.51]), while no differences were observed in the Neutral (*t*_297_ = -1.43, *p*_FDR_ = .288, *d* = -0.30, CI [-0.70, 0.12]) or Joy (*t*_297_ = -0.68, *p*_FDR_ = .363, *d* = -0.14, CI [-0.56, 0.27]) states. In addition, women significantly discounted more steeply when in the fearful condition (0.32 ± 0.22) as compared to Joy (0.42 ± 0.25) (*t*_297_ = -2.51, *p*_FDR_ = .039, *d* = -0.45, CI [-0.80, -0.10]), but not compared to the Neutral condition (0.42 ± 0.25) since this significant difference did not survive after multiple testing correction (*t*_297_ = -2.00, *p*_FDR_ = .100, *d* = -0.36, CI [-0.71, -0.003]). Regarding the comparisons between the male groups, we found no statistically significant differences among the three experimental conditions, suggesting that the males’ DD was not influenced by the induced emotions (Fear vs. Neutral and Joy conditions: *p*_FDR_ = .289 and *p*_FDR_ = .277 respectively; Neutral vs. Joy *p*_FDR_ = .797).

### Affective experience according to emotional condition and gender

#### Affective slider—Arousal

**Fig 4:**
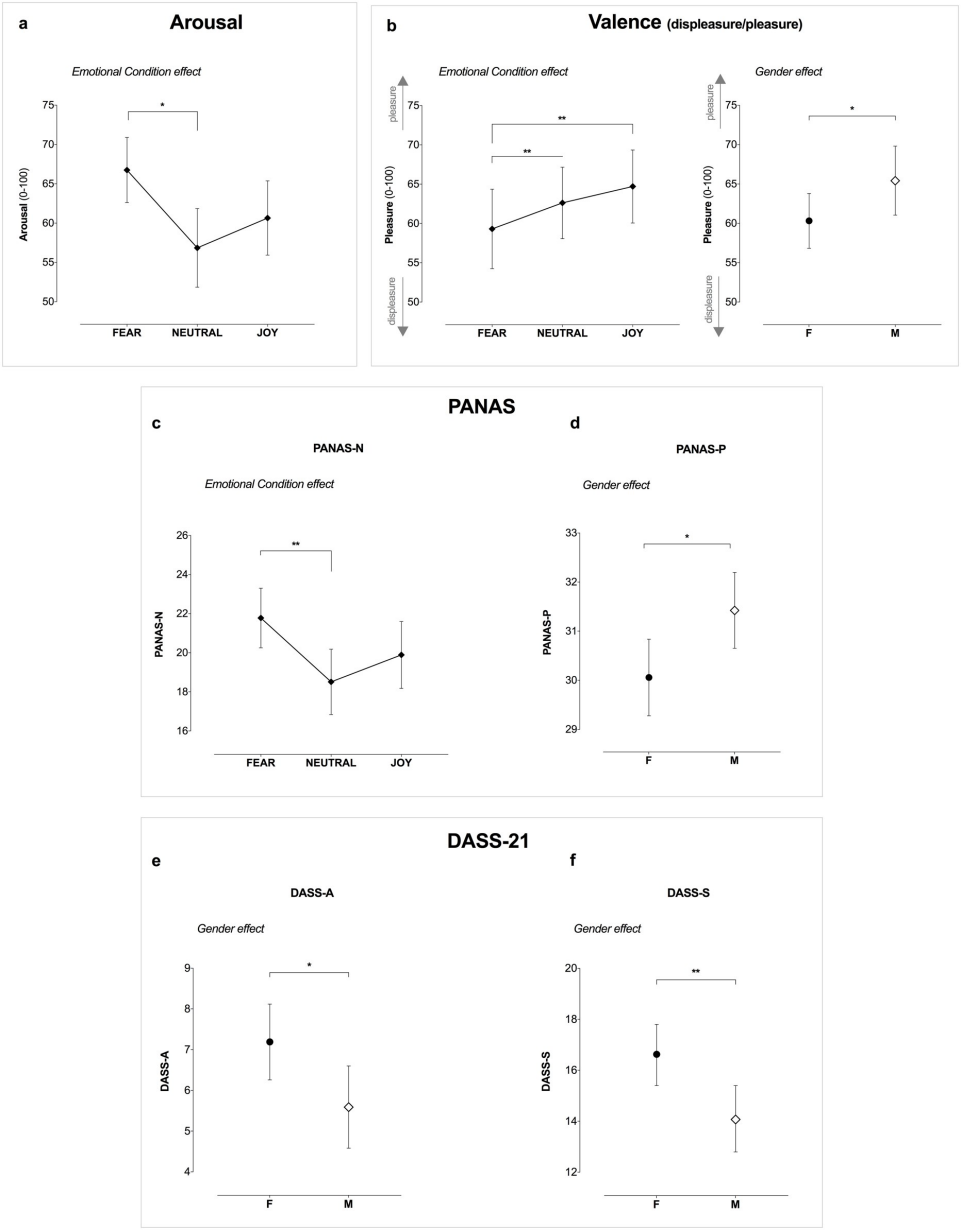
Statistically significant gender or induced emotion effects on the affective experience, as measured by (a) arousal, (b) valence and by the Positive and Negative Affect Schedule (PANAS) with (c) its negative (PANAS-N), and (d) positive (PANAS-P) subscales. As well as on the psychological dimensions as measured by Depression Anxiety Stress Scales-21 (DASS-21) assessing presence of anxiety (DASS-A) (e) and stress (DASS-S) (f). ** p < .005; * p < .05.

We found an effect of the Emotional Condition on the arousal levels (*F*_2,302_ = 4.07, *p* = .018, η_p_^2^ = .03), with higher levels of arousal in the fearful condition as compared to the Neutral one (*t*_302_ = 2.83, *p*_FDR_ = .015, *d* = 0.41, CI [0.12,0.69]; Fig 4a), while no significant differences were observed between the other experimental conditions. Further, no Gender (*F*_1,302_ = 0.11, *p* = .746) and Gender × Emotional Condition interaction effects (*F*_2,302_ = 0.07, *p* = .930) were observed.

#### Affective slider—Valence

Concerning the valence level, we observed a main effect of Emotional Condition induced by the stimuli (*F*_2,301_ = 6.48, *p* = .002, η_p_^2^ = .04) as well as of Gender (*F*_2,301_ = 4.00, *p* = .046, η_p_^2^ = .01; Fig 4b). Namely, post hoc tests revealed that a more positive valence was reported in the Joy condition (*t*_301_ = -2.94, *p*_FDR_ = .005, *d* = -0.43, CI [-0.71, -0.14]) and the Neutral one (*t*_301_ = -3.25, *p*_FDR_ = .003, *d* = -0.47, CI [-0.76, -0.18]) as compared to Fear. Instead, regarding Gender effect, males overall reported a more positive valence than females (*t*_301_ = -2.00, *p* = .046, *d* = -0.24, CI [-0.47, 0]), regardless of the experimental condition. Whereas no Gender × Emotional Condition interaction effect was observed (F_2,301_ = 0.49, p = .614).

#### PANAS

Analyses about its negative component (PANAS-N) showed a main effect of Emotional Condition (*F*_2,301_ = 6.07, *p* = .003, η_p_^2^ = .04; Fig 4c), while no Gender (*F*_1,301_ = 0.62, *p* = .431) or Gender × Emotional Condition interaction effects were observed (*F*_2,301_ = 2.10, *p* = .125). In particular, we found a higher level of Negative emotional state in the Negative condition than in the Neutral one (*t*_301_ = 3.48, *p*_FDR_ < .003, *d* = 0.51, CI [0.22,0.80]; Fig 4c), but no further differences were observed between the other conditions. About the PANAS-P, we found only a Gender effect (*F*_2,301_ = 5.56, *p* = .019, η_p_^2^ = .02; Fig 4d) with men reporting a more positive emotional state, regardless of the conditions. No Gender × Emotional Condition interaction effect was observed (F_2,301_ = 0.85, p = .848).

### Psychological outcomes according to emotional condition and gender

ANOVA results revealed a main effect of gender on the DASS-21(*F*_2,302_ = 4.76, *p* = .030, η_p_^2^ = .02), wherein the female group reported overall higher levels of general distress. Whereas no Emotional Condition (*F*_2,302_ = 0.45, *p* = .640) as well as Gender × Emotional Condition (*F*_2,302_ = 0.66, *p* = .520) effects were observed.

Analyzing the DASS-21 subtests, this between-group difference was mainly driven by anxiety and stress, wherein the female group reported higher DASS-A (*F*_2,302_ = 4.86, *p* = .028, η_p_^2^ = .02; Fig 4e) and DASS-S scores (*F*_2,302_ = 6.98, *p* = .009, η_p_^2^ = .02; Fig 4f) than men, respectively. No differences were observed in the depression subscale. Finally, following ANOVA analysis on impulsivity levels (BIS-11), we found no significant main effects of gender and emotional condition, nor interaction.

### Prevalence of psychological disorders

**Fig 5:**
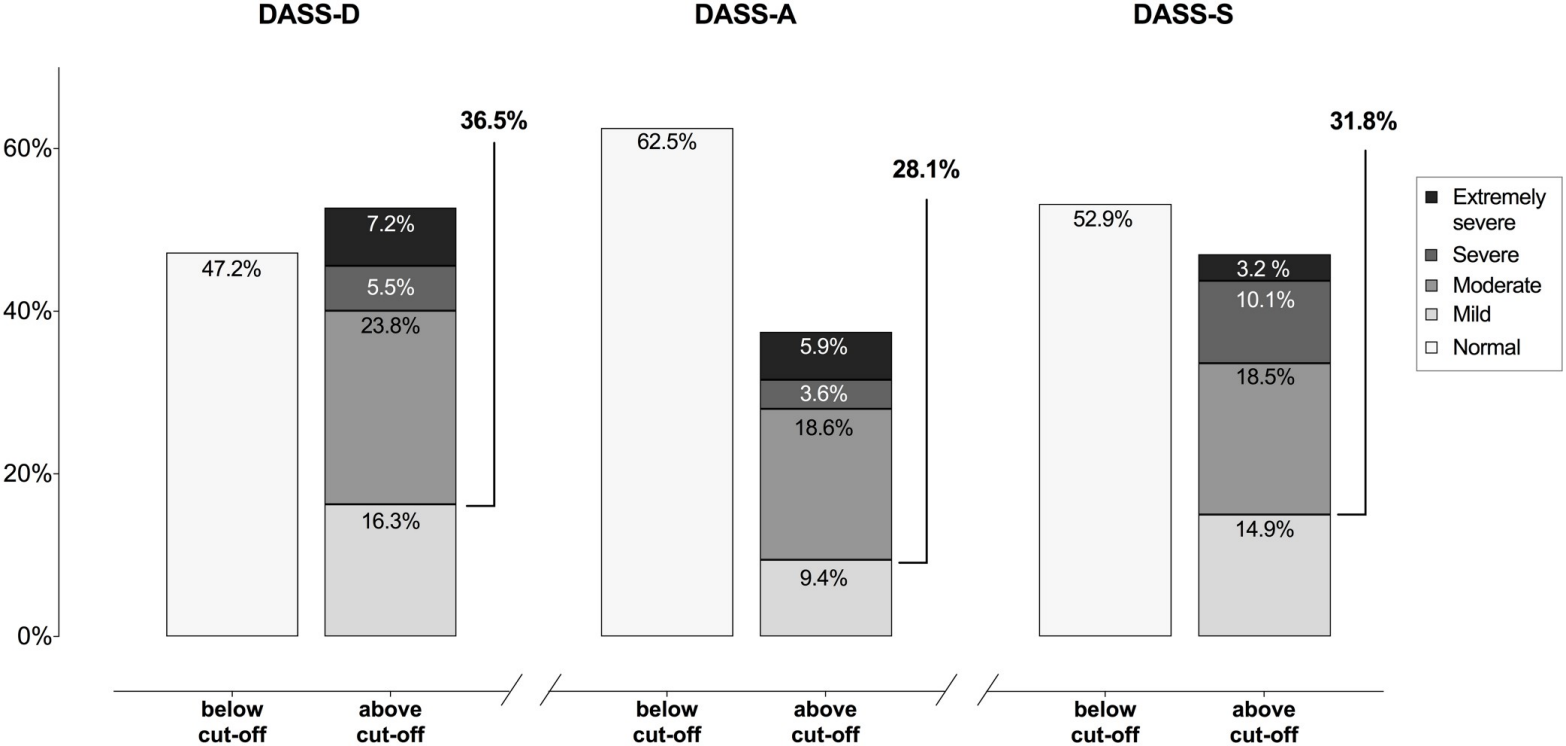
Prevalence of depression (DASS-D), anxiety (DASS-A), and stress (DASS-S) according to the symptoms’ severity.

As shown in Fig 5, the prevalence of participants reporting symptoms above the clinical cut-offs [83] was about 53 percent for depression (n = 162), 37 percent for anxiety (n = 115), and 47 percent for stress (n = 144). The average score for depression, anxiety and stress was 11.35 (*SD* = 9.16), 6.61 (*SD* = 6.28) and 15.69 (*SD* = 8.23), respectively. The DASS-21 total score, measuring general distress, was about 33.65 (*SD* = 20.67).

## Discussion

The present study provides compelling evidence that gender has a crucial role in the interplay between emotions and intertemporal decision-making, with gender and emotions having an interacting effect that can differently shape DD. Our key finding was that, when experiencing unpleasant emotions, particularly fear, women exhibited a steeper discounting of future rewards compared to men. Women were also more likely to prefer immediate rewards when they were in a negative emotional state rather than in a positive one, whereas men showed an opposite intertemporal tendency compared to women, preferring delayed rewards when they were in a fearful state. Furthermore, while women’s discounting rate varies based on the emotional state, men’s choices on monetary rewards were not significantly affected by the emotional state.

As such, our study provides the first evidence that fear can trigger different implicit goals and intertemporal choices as a function of the gender.

Previous decision-making studies have strongly highlighted gender difference in reward-sensitivity and in information processing, demonstrating the existence of distinctive gender decision-making styles. Males show a tendency towards maximizing long-term benefits by using a more global-selective information processing style—whereas females are more prone to maximize either immediate or long-term rewards in different situations by using more detailed-comprehensive processors of information [43, 49, 65]. This leads us to conclude that when individuals experience negative or fearful emotional states, gender-specific decision-making strategies come into play. Specifically, in situations marked by uncertainty, females tend to display risk-averse and uncertainty-avoidant behaviors by favoring immediate rewards. Conversely, males’ inclination towards larger rewards may make them more willing to tolerate uncertainty and risk to ultimately secure a larger gain. In other words, the disparities in reward sensitivity and associated strategies between males and females appear to interact with their emotional states. We can speculate that states with a high uncertainty (commonly associated with a feeling of fear) could affect how much risk people are willing to tolerate: females show a lower tolerance for risk and higher tendency for immediate/safe rewards, while males are more prone to risk for their primary goal of obtaining larger rewards. Notably, these observations converged with evidence from several decision-making studies (e.g., see [65]).

### An evolutionary perspective

Considering that emotions serve adaptive functions [86], a potential explanation of our results can emerge from the evolutionary framework. Our finding that females are more oriented to choose immediate reward when in a fearful condition seems to fit within this framework. Indeed, in a fearful condition, the immediate reward can have an adaptive function of immediate self- and offspring-preservation. Under stressful and fearful environmental situations, future rewards (e.g., food) could also be more unpredictable and uncertain than the immediate ones, therefore opting for a future reward could put the offspring in a risky and unsafe condition. In this regard, the differences in DD between males and females would reflect the differences in evolutionary strategies.

Aligned with this perspective, an established theory by Taylor and co-workers (2000) posited that females, when facing stress/fearful situations, are more prone to activate a ‘tend-and-befriend’ response, rather than the typical ‘fight-or-flight’ behavior, supporting the idea that males and females face fearful situations in a very different manner [87]. As a matter of fact, the ‘fight-or-flight’ response can put females and their offspring at risk, while tending-and-befriending is a more adaptive response to stress/fearful situations, especially during pregnancy or with new-borns, aimed at protecting self and offspring. Following this reasoning, when experiencing fear, females’ preference for immediate/certain rewards can be considered as part of their tending behavioral pattern, which requires prompt nurturing activities to preserve the safety as well as to reduce the current stressful feeling [86].

### Emotion-regulation strategies

An alternative perspective to further understand our current findings can emerge by considering emotion-regulation strategies, as gender can affect the extent and the modality in which emotions are regulated [86, 88]. Previous investigations showed that females tend to use coping strategies, aimed at changing the emotional responses of a stressful situation, whereas men use more problem-focused or instrumental methods to handle stressful situations [89–92]. In support of this view, choosing the immediate reward can serve as a regulatory function, with immediate gratification resulting in an alleviation of the negative affect.

As such, DD is thought to rely on two separate processes: an emotional/visceral versus a logical/rational process [93]. The degree to which one chooses the emotionally relevant response (i.e., the immediate reward) can possibly be determined by the amount of regulatory necessity, which is driven by the relationship between the emotional state (i.e., experienced fear) and the individual regulatory tendencies–wherein, gender plays a crucial role.

This perspective seems to support our data. In fact, in the context of an equivalent fear perception between males and females, as assessed by the self-reported negative affect (i.e., PANAS-N), opposite tendencies on intertemporal choices can be explained by adopting different emotion regulation strategies. Namely, females are more oriented to change their negative emotional state and thus to choose an immediate reward, whereas men with a problem-focused coping strategy will be more prone to choose the larger-later reward. Hence females, when in fearful state, may be more prone to select the objectively less valued-immediate reward [94] to reduce negative emotional states [95].

Of note, we found no interaction effect between Gender and the Emotional Conditions in the negative self-reported affective state (PANAS-N) as well as in the other outcomes assessing the emotional experience (i.e., PANAS-P and the Affective slider). Hence, although we found gender/sex differences in the self-reported emotional experience–intrinsic to women and men–these differences were not modulating the experimental condition, supporting the robustness of our findings observed in the intertemporal decision-making.

Furthermore, higher levels of anxiety and stress were reported by the female group. This is unsurprising given that findings from epidemiological studies have repeatedly reported a higher prevalence of affective disorders in women [90], across the life span [86]. Since higher levels of anxiety, depression and stress were found to be predictive of steeper discounting rates [15], it is possible to hypothesize a cumulative effect of the fearful task condition on the negative mood experienced largely by women. Of note, our data collection occurred during the COVID-19 pandemic, between May and July, 2021. Despite the relaxation of the preventive measures in Italy during this timeframe, we cannot exclude the presence of long-term pandemic effects on psychological outcomes [96]. Indeed, in the current study, psychological disorders prevalence exceeded the pre-pandemic rates [97], and these high levels are expected to persist over time [98].

Among the measures to objectively assess the emotional experience, we found that the fearful condition was characterized by higher levels of arousal, of negative valence and negative affect (PANAS-N) than the other emotional conditions (neutral state/joy). Conversely, movie clips with a positive emotional valence were characterized by higher levels of positive valence as compared to the negative ones, but not to the neutral conditions. Hence, these data seem to support the validity and effectiveness of the emotion manipulation in particular of the fearful condition, but not of the positive one.

Contrary to our initial hypothesis, we did not observe a preference for delayed larger rewards after inducing a positive state of happiness or joy. This led individuals to make relatively optimistic risk assessments and choices [1]. However, we cannot exclude that this was due to an ineffective positive emotion induction. Indeed, other positive and arousing stimuli, such as erotic movie clips [58], could differently shape intertemporal choices as a function of gender. In fact, Wilson and Daly (2004) demonstrated that in their sample, men discounted the future more steeply after considering the appealing pictures of pretty women, but higher discounting rates were not observed in females after viewing pictures of attractive men [44]. Hence, we believe that future studies using more ecological stimuli, such as erotic film clips, will be necessary to better explore and corroborate this evidence.

A final observation is that we did not find an association between intertemporal choices and impulsivity, as measured by the BIS-11 scale. This result adds to the view that other mechanisms, such as reward sensitivity and decision-making strategies [65], interacting with gender and emotional states, can better account for different discounting behaviors.

### Strength, limitations and future directions

The major shortcoming of the current study is that our sample was gender-imbalanced, with a majority of female participants–about the 63 percent. However, we tested the robustness of our findings through random undersampling analyses, which corroborated the results obtained in the total sample. This seems to be a common issue among web-based surveys, indeed although online recruitment facilitates diversity (with participants varying in geographical location, socioeconomic status, educational level, and age). On the other side, it has been reported [99] that female participants are frequently overrepresented in web-based surveys, possibly due to gender differences in internet use patterns [100].

Further, in our sample we found differences in psychological disorders prevalence between males and females. This finding was expected, as it is strongly supported by previous evidence reporting higher rates of anxiety, distress and depression in women rather than men [90, 97]; however, we cannot exclude that this could have possibly amplified some observed effects following the induction of distinct emotions, such as fear. To this end, the DASS-21 total score was included as a nuisance variable in the statistical analyses to control the different ‘baseline’ level related to gender. Another methodological limitation of our study is that the standardized movies differed in terms of arousal levels between positive and negative valences, however arousal was included in the analyses as covariate to control its potential confounding effect [101]. Using erotic movies, which are comparable in terms of arousal to the fear movies [58], can be a solution for future study designs. However, for ethical reasons, using erotic movies as experimental condition was unfeasible given the web-based nature of the study.

According to the affective experience evaluation, the positive emotion induction was unsuccessful, since this did not differ from the neutral control condition. Although the affective experience assessment did not specifically evaluate the discrete emotions (e.g., fear), but more general tools were adopted (i.e., PANAS for the negative/positive valence).

Lastly, another potential limitation is the use of a hypothetical money reward, instead of a real one, which could have reduced the participants’ engagement in the decision task as compared to a real situation with actual gains/losses. Although previous evidence extensively demonstrated an equivalence between real and hypothetical monetary rewards [75, 102].

Our study has also come strengths. The emotion induction was based on a standardized and validated dataset of emotional movie clips (‘EMOVIE’) to elicit discrete emotions with a more ecological and clear-cut approach [58]. The psychological outcomes and affective states were assessed using self-reported standardized measures (i.e., DASS-21, BIS-11, PANAS), which can be useful for the between-studies comparison. Further, a computerized hypothetical monetary DDT was adopted, which allows an objective assessment of different decision-making tendencies.

## Conclusions

The present study provides theoretical insights into the role of gender when experiencing distinct emotions, such as fear and joy, during intertemporal choices. In particular, we found an increased tendency to opt for immediate rewards by women when in a fearful condition—as opposed to men. Also, women were more prone to choose immediate rewards when in a fearful condition, than when in a positive state of joy/happiness. By contrast, when in a positive or neutral emotional state, females and males did not differ.

Our findings extend the literature by revealing the significance of considering gender as an interacting factor between emotions and decision-making. This evidence suggests important psychological and sociological implications, as we could hypothesize that when women are facing fearful situations, they are more impatient and impulsive, given that fear, unlike happiness/joy, induces females to be more ‘myopic’ to–greater–future gains in return of instant gratification. If so, this finding can be relevant in the context of financial decision-making or consumer behaviors. Strong feeling of fear in real-life can be expected to show far greater effects on many intertemporal decisions, possibly leading women–experiencing a fearful condition–to exacerbate their financial hardship by making intertemporal decisions that favor an immediate consumption rather than a wiser and warranted one.

On the other hand, this female behavior could be the result of evolution, which leads females to prefer immediate–but safer–rewards rather than future–but uncertain–ones.

To conclude, we argue that when studying emotions and decision-making, both gender differences should be considered, as this perspective may improve our understanding and predict the effects on financial, health and social decisions; given that intertemporal choices underlie a variety of decisions in our daily life. As an ultimate goal, learning more about this interplay’ can be relevant to develop techniques that can help individuals in making more optimal intertemporal decisions in their real-life, avoiding unhealthy/maladaptive behaviors.

## Supporting information

S1 Checklist STROBE statement—Checklist of items that should be included in reports of observational studies. (PDF)

S1 File (PDF)
