# Supplementary material for: Gender differences in the effects of emotion induction on intertemporal decision-making
Source: PLoS One. 2024 Mar 20;19(3):e0299591. doi: 10.1371/journal.pone.0299591 (PMC10954116; doi:10.1371/journal.pone.0299591)

SUPPLEMENTARY RESULTS

Participant sociodemographic and lifestyle related features

Following the random undersampling, we obtained a balanced dataset for gender in each condition [female/male: Fear (41/41), Neutral (36/36) and Joy (36/36)]. The subsample (n = 226) sociodemographic and lifestyle characteristics are shown in Supplementary Table 1.

Gender and emotional valence effects on delay discounting

ANCOVA results showed a significant Gender × Emotional Condition interaction on DD ($F_{2,215}$ = 4.20, p = .016, η_p^2 = .04; eFigure 1). Post-hoc analysis revealed that in the Fear condition, women (0.29 ± 0.23) showed a statistically significant higher DD than men (0.54 ± 0.25) (t_{216} = -4.61, p_{FDR} < .001, d = -1.02, CI [-1.47, -0.58]), while no differences were observed (as compared to men) in the Neutral or Joy states. In addition, analyses within the female group highlighted that women significantly discounted more steeply when in the Fear condition (0.29 ± 0.23) as compared to Joy (0.43 ± 0.25) (t_{216} = -2.67, p_{FDR} = .033, d = -0.62, CI [-1.07, -0.16]), but not compared to the Neutral condition (0.39 ± 0.25) since it did not reach significance after multiple testing correction (t_{216} = -1.85, p_{FDR} = .165, d = -0.43, CI [-0.88, 0.03]). Regarding the comparisons within the male group, we found no statistically significant differences between the three experimental conditions, suggesting that the males' DD was not influenced by the induced emotions.

Affective experience according to emotional valence and gender

Arousal. Following ANOVA model, we found no statistically significant difference with regard to Emotional Valence, Gender and interaction effects.

Pleasure. Regarding the pleasure level, ANCOVA results highlighted a main effect of Emotional Valence induced by the stimuli ($F_{2,219}$ = 3.21, p = .042, η_p^2 = .03) as well as of Gender ($F_{2,219}$ = 4.15, p = .043, η_p^2 = .04; eFigure 2a). However, following post hoc tests these differences were no more statistically significant. Namely, a tendency for higher levels of pleasure were reported in the Positive (t_{219} = -2.12, p_{FDR} = .081, d = -0.36, CI [-0.69, -0.04]) and the Neutral condition (t_{219} = -3.25, p_{FDR} = .052, d = -0.35, CI [-0.67, -0.02]) as compared to the Negative one. Instead, regarding Gender effect, the male group reported higher levels of pleasure than females (t_{219} = -2.04, p = .043, d = -0.27, CI [-0.54, -0.007]), independent of the conditions.

PANAS. The affective experience was further explored with PANAS. Analyses about its negative component (PANAS-N) showed a main effect of Emotional Valence ($F_{2,219}$ = 3.52, p = .032, η_p^2 = .03; eFigure 2b), while no Gender ($F_{1,219}$ = 1.32, p = .251) or interaction effects were observed ($F_{1,219}$ = 1.64, p = .197). In particular, we found a higher level of Negative emotional state in the Negative condition than in the Neutral one (t_{219} = 2.64, p_{FDR} = .003, d = 0.43, CI [0.11,0.75]; eFigure 2b), but no further differences were observed between the other conditions. About the PANAS-P, we found only a Gender effect ($F_{2,219}$ = 6.11, p = .014, η_p^2 = .03; eFigure 2c) with men reporting a more positive emotional state, independent of the conditions.

Psychological outcomes according to emotional valence and gender

We found no statistically significant main effects of gender and emotional valence, nor interaction in the DASS-21 as well as in impulsivity levels (BIS-11).

Prevalence of psychological disorders

The prevalence of participants reporting symptoms above the clinical cut-offs (Lovibond & Lovibond, 1996) was about 50 percent for depression (n = 113), 37 percent for anxiety (n = 84), and 44 percent for stress (n = 99). The average score for depression, anxiety and stress was 10.69 (*SD* = 8.72), 6.70 (*SD* = 6.47) and 15.26 (*SD* = 8.14), respectively. The DASS-21 total score, measuring general distress, was about 32.66 (*SD* = 20.41).

Supplementary Table 1 Total sample (N = 226) sociodemographic features.

		Group	n	%
**Age**	**Mean (SD):** 33.40(14.21)	18-24	94	41.59
		25-40	73	32.30
	**Min–max:** 18–74	>40	59	26.11
**Gender**		Female	113	50
		Male	113	50
**Education**		Middle school	11	4.90
		High school	104	46.00
		Bachelor’s degree	67	29.60
		Master’s degree	31	13.70
		PhD/ postgraduate	13	5.80
**Emotional movie clip**		Negative	82	36.30
		Neutral	72	31.90
		Positive	72	31.90

eFigure 1 Gender and induced emotion effect on delay discounting, as measured by the area under the curve (AUC) of the delay discounting task (DDT). Error bars represent the 95% confidence interval of the mean. *** p < .001; * p < .05.

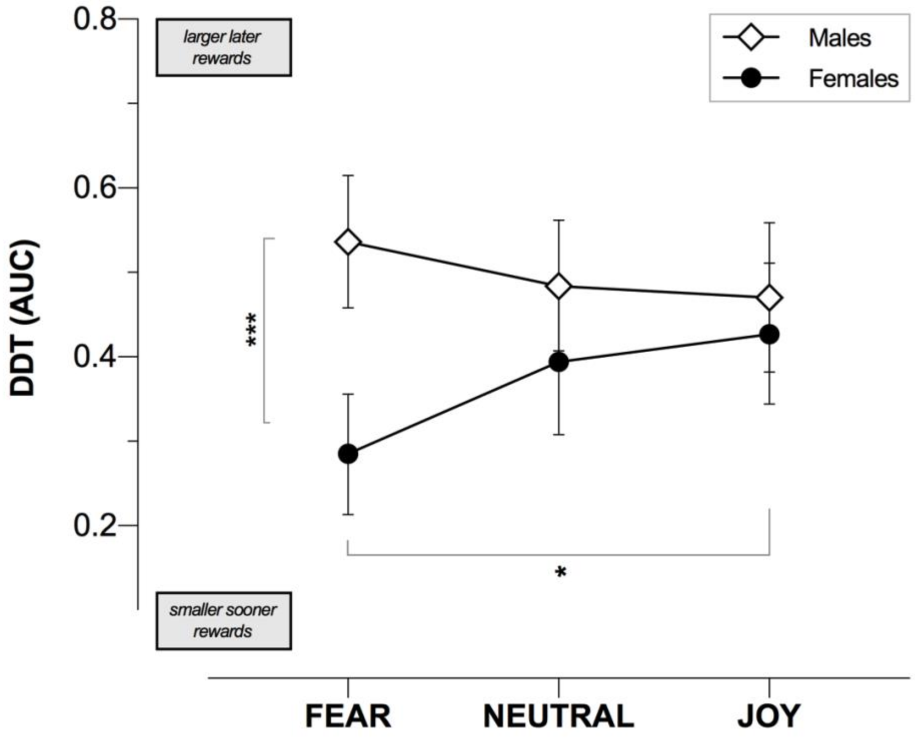

eFigure 2 Statistically significant gender or induced emotion effects on the affective experience, as measured by (a) pleasure and by the Positive and Negative Affect Schedule (PANAS) with (b) its negative (PANAS-N), and (c) positive (PANAS-P) subscales. ** p < .005; * p < .05

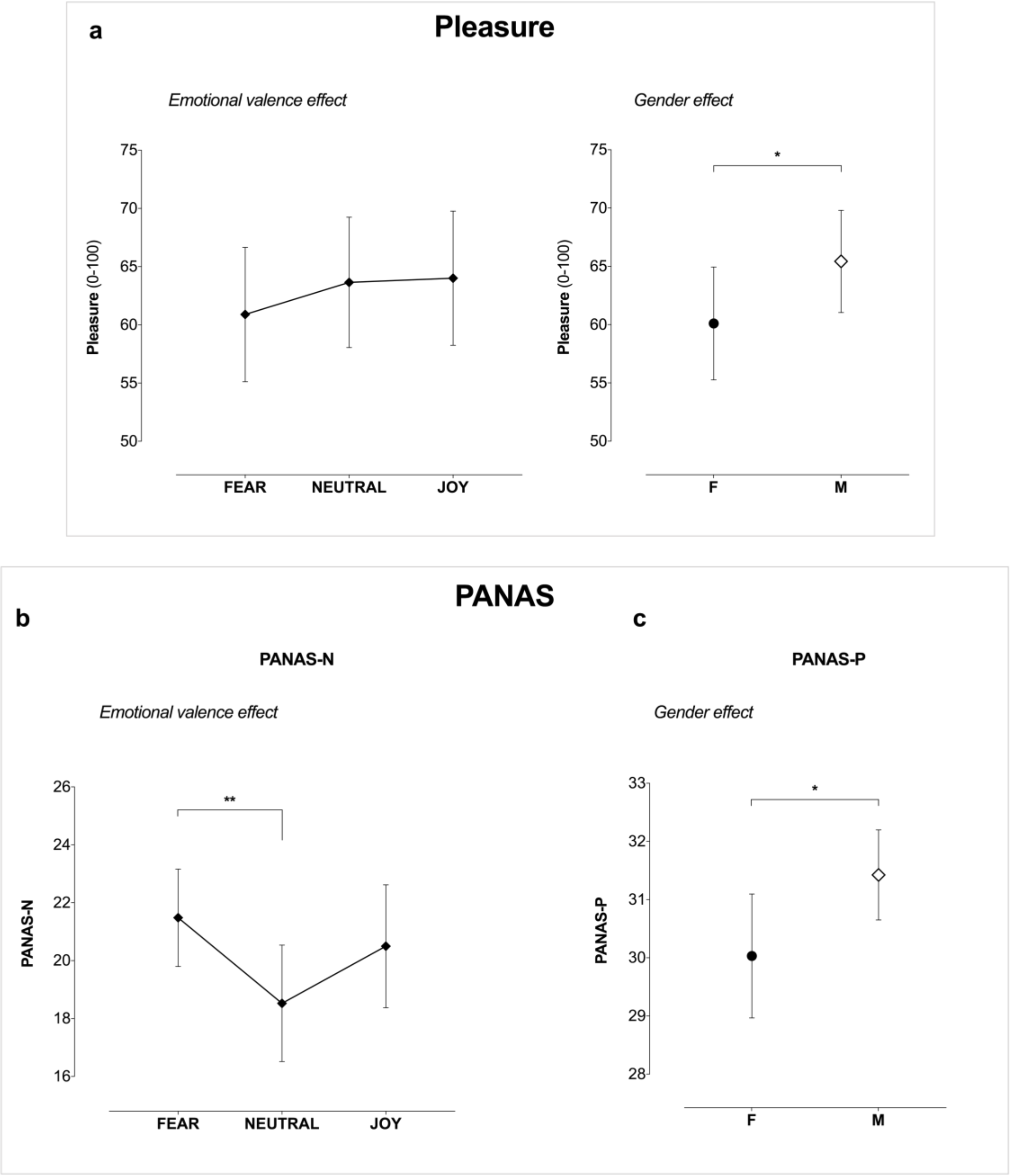

Supplement: S1 File — (PDF) [file pone.0299591.s002.pdf]
